# The Differential Roles of T Cells in Non-alcoholic Fatty Liver Disease and Obesity

**DOI:** 10.3389/fimmu.2019.00082

**Published:** 2019-02-06

**Authors:** Mikhaïl A. Van Herck, Jonas Weyler, Wilhelmus J. Kwanten, Eveline L. Dirinck, Benedicte Y. De Winter, Sven M. Francque, Luisa Vonghia

**Affiliations:** ^1^Laboratory of Experimental Medicine and Pediatrics, Division of Gastroenterology and Hepatology, University of Antwerp, Antwerp, Belgium; ^2^Department of Gastroenterology and Hepatology, Antwerp University Hospital, Antwerp, Belgium; ^3^Department of Endocrinology, Diabetology and Metabolism, Antwerp University Hospital, Antwerp, Belgium

**Keywords:** non-alcoholic fatty liver disease, non-alcoholic steatohepatitis, obesity, type 2 diabetes mellitus, T helper cells, regulatory T cells, cytotoxic T cells, natural killer T cells

## Abstract

Non-alcoholic fatty liver disease (NAFLD) constitutes a spectrum of disease states characterized by hepatic steatosis and is closely associated to obesity and the metabolic syndrome. In non-alcoholic steatohepatitis (NASH), additionally, inflammatory changes and hepatocellular damage are present, representing a more severe condition, for which the treatment is an unmet medical need. Pathophysiologically, the immune system is one of the main drivers of NAFLD progression and other obesity-related comorbidities, and both the innate and adaptive immune system are involved. T cells form the cellular component of the adaptive immune system and consist of multiple differentially active subsets, i.e., T helper (Th) cells, regulatory T (Treg) cells, and cytotoxic T (Tc) cells, as well as several innate T-cell subsets. This review focuses on the role of these T-cell subsets in the pathogenesis of NAFLD, as well as the association with obesity and type 2 diabetes mellitus, reviewing the available evidence from both animal and human studies. Briefly, Th1, Th2, Th17, and Th22 cells seem to have an attenuating effect on adiposity. Th2, Th22, and Treg cells seem to decrease insulin resistance, whereas Th1, Th17, and Tc cells have an aggravating effect. Concerning NAFLD, both Th22 and Treg cells appear to have an overall tempering effect, whereas Th17 and Tc cells seem to induce more liver damage and fibrosis progression. The evidence regarding the role of the innate T-cell subsets is more controversial and warrants further exploration.

## Introduction

Non-alcoholic fatty liver disease (NAFLD) is characterized by the presence of hepatic steatosis without causes for secondary hepatic fat accumulation and is comprised of a spectrum of multiple disease states, going from non-alcoholic fatty liver (NAFL) to non-alcoholic steatohepatitis (NASH) to NAFLD-related advanced liver disease with all its complications. The presence of steatosis in conjunction with both lobular inflammation and ballooning of the hepatocytes—a histological sign of hepatocellular damage—defines NASH, which may be accompanied by progressive fibrosis. When these histological trademarks of NASH are not both present, the term NAFL is used (1, 2). The distinction between these two disease states is of utmost importance, as NASH represents a more active side of the spectrum with a more rapid progression to advanced liver disease (1). A close relationship has been highlighted between NAFLD and the metabolic syndrome, clustering visceral overweight, dyslipidaemia, insulin resistance, and arterial hypertension (2). This has led to the generally accepted idea that NAFLD is the hepatic manifestation of the metabolic syndrome (3). Obesity, in particular, is the most common and well-documented risk factor for NAFLD, and in itself represents an enormous public health issue, taking on epidemic proportions and predisposing for a myriad of comorbidities, including type 2 diabetes mellitus (DM2), cardiovascular disease and malignancy. Moreover, in NAFLD, obesity was shown to be an independent predictor of fibrosis progression, development of NASH, and mortality (4). As is the case for obesity and DM2, the prevalence of NAFLD is increasing in many Western countries, reaching 25–30% in the general population and 42–72% in patients affected by DM2 (1, 5). Importantly, NAFLD has become one of the three major causes of cirrhosis, is associated with the occurrence of hepatocellular carcinoma—even in a non-cirrhotic state—and is an independent risk factor for cardiovascular disease, underlining the vast burden on public health (6–8). Additionally, it has been suggested that NAFLD itself is implicated in the pathophysiology of DM2, establishing a multidirectional disease state (9).

The pathogenesis of NASH is complex and involves a crosstalk between various metabolically active tissues. A multitude of processes may contribute to liver damage and inflammation, including insulin resistance, hepatic fatty acid accumulation, oxidative stress, mitochondrial dysfunction, and a systemic proinflammatory state. These multiple hits probably take place in parallel, rather than consecutively, hence the term “multiple parallel hits hypothesis” (10, 11). Importantly, NAFLD is no longer considered an exclusively hepatic disease, as multiple other organ systems participate in the pathogenesis of liver inflammation (10). Two key players are especially of interest, being on the one hand the gut, through dysregulation of the microbiome, and on the other hand the adipose tissue (10, 12, 13). Considering the latter, obesity predisposes to adipose tissue hypertrophy, resulting in increased lipolysis, as well as adipose tissue inflammation. Collectively, these features result in adipose tissue dysfunction, which is characterized by an impaired adipokine secretion and a higher release of free fatty acids, and is critically involved in the pathogenesis of obesity-related pathologies, including insulin resistance and NAFLD (14). In the context of NAFLD, it has been estimated that 60% of the hepatic triglycerides originates from adipose tissue lipolysis (15). The resulting increased availability of lipids then causes hepatocellular injury, a concept known as lipotoxicity, through endoplasmic reticulum stress, oxidative stress and mitochondrial impairment (4). Jointly, these factors are implicated in the development of hepatic inflammation, fibrosis and eventually tumorigenesis. Cytokines, adipokines and cells of the innate and adaptive immune system favor a crosstalk between the gut, adipose tissue and the liver and are therefore key drivers of NASH pathogenesis (10, 13).

**Figure 1:**
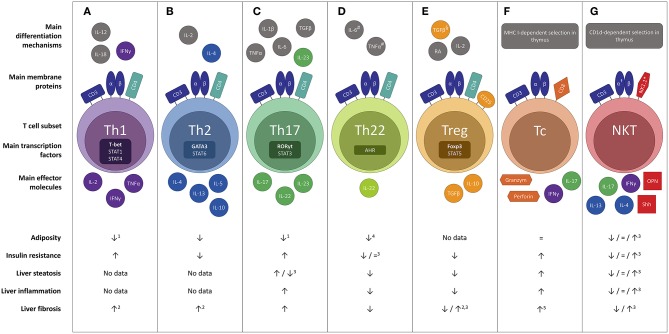
Overview of the differential effects of the main T cell subsets in the pathophysiology of NAFLD : **(A)** Th1, **(B)** Th2, **(C)** Th17, **(D)** Th22, **(E)** Treg, **(F)** Tc and **(G)** NKT. Evidence was included in the figure exclusively when the effect was demonstrated in gain-of-function or loss-of-function experiments. ^#^In the absence of TGF-β. ^§^In the absence of IL-6.^†^Invariant NKT cells express a semi-invariant TCRα chain (Vα14Jα18 in mice and Vα24Jα18 in humans). *In humans the natural killer cell marker CD56 is sometimes used. Neither CD56 nor NK1.1 is considered specific enough to characterize NKT cells. ^1^Yet more adipose tissue inflammation. ^2^Not proven in the context of NAFLD. ^3^Conflicting evidence. ^4^Upon administration of high, non-physiologic doses of IL-22. ^5^To a minor extent. AHR, aryl hydrocarbon receptor; CD, cluster of differentiation; Foxp3, forkhead box P3; IL, interleukin; NKT, natural killer T cell; OPN, osteopontin; RA, retinoic acid; RORγt, RAR-related orphan receptor γt; Shh, Sonic Hedgehog; T-bet; Tc, cytotoxic T cell; Th1, T helper 1; Th2, T helper 2; Th17, T helper 17; Th22 T helper 22; Treg, regulatory T cell.

Both the innate and the adaptive immune system are involved in obesity-related adipose tissue inflammation and the pathogenesis of NAFLD. At the level of the adipose tissue, obesity causes an early accumulation of T cells, as discussed below, subsequently altering the predominant phenotype of the adipose tissue macrophages (ATMs) to a more proinflammatory state. Importantly, this key event is thought to precede the above-described adipocyte hypertrophy present in obesity (16). Cells of both the innate and adaptive immune system will then form clusters surrounding adipocytes undergoing apoptosis in characteristic “crown-like structures” (14, 17). In NAFLD, macrophages, i.e., Kupffer cells and monocyte-derived macrophages, are key players considering the innate immunity (18). Following the development of steatosis, hepatocytes and Kupffer cells secrete chemotactic agents, including chemokine C-C motif ligand 2 (CCL2, also referred to as monocyte chemotactic protein-1, MCP-1), thus increasing the liver macrophage pool through monocyte infiltration (18, 19). Subsequently, a positive feedback mechanism is established through secretion of large amounts of proinflammatory cytokines, including interleukin- (IL) 1β and tumor necrosis factor (TNF) α, driving liver steatosis, inflammation, and fibrosis (19). Other components of the innate immune system, including natural killer cells and dendritic cells (DCs), also contribute to NAFLD pathogenesis, respectively, by cytotoxic effects and by production of proinflammatory and anti-inflammatory cytokines (18–20). With respect to the adaptive immunity, particularly T cells are involved in NAFLD. T cells constitute the cellular component of the adaptive immune system, as opposed to B cells that represent the humoral component. Specific T-cell effector functions are induced through recognition of antigens presented on the major histocompatibility complex (MHC) class I or II of antigen presenting cells. Antigen recognition is achieved through the specialized T-cell receptor (TCR), which is composed of an α and β protein in the majority of T cells (hence αβ T cells), and the CD3 complex, an essential co-receptor, which is present on all T cells and is commonly used in their characterization. Moreover, T cells can be subdivided in multiple subsets with differential physiologic functions, most importantly CD8^+^ cytotoxic T (Tc) cells and various CD4^+^ T helper subsets, as well as several subsets of T cells belonging to the innate rather than the adaptive immune system, including natural killer T (NKT) cells, γδ T cells and mucosal-associated invariant T (MAIT) cells (21). These phenotypically diverse subsets can be characterized, most commonly by flow cytometry, using specific markers, including membrane proteins, transcription factors and cytokines. This review will focus on the role of these functionally diverse T-cell subsets in the pathogenesis of NAFLD, as well as their concurrent involvement in obesity and insulin resistance (Figure 1).

## T Helper Cells

T helper cells are key regulators of proinflammatory and anti-inflammatory immune processes and are characterized by membrane expression of CD4 (20). The CD4 molecule is a co-receptor to the TCR that ensures specificity for antigens presented by the MHC II (21). T helper cells derive their name from their crucial role in supporting the effector functions of B cells, Tc cells and phagocytes, and are further characterized by their function and major cytokine secretion (18). It is important to note, however, that a large degree of plasticity exists between the various T helper subsets, as distinct lineages may change their phenotype in the presence of the appropriate stimuli (22).

### T Helper 1 Cells

T helper 1 (Th1) cells are proinflammatory cells that express the transcription factor T-bet and produce interferon (IFN) γ, IL-2, and TNFα (Figure 1A). The critical cytokines for Th1 differentiation are IL-12 and IFNγ itself, through signal transducer and activator of transcription (STAT) 4 and STAT1 activation (23). Physiologically, Th1 cells play an important role in the cellular component of the adaptive immune system, especially in the defense against intracellular pathogens (24).

It has been well-established that Th1 cells are involved in adipose tissue inflammation associated with obesity-related pathologies (Table 1). In a high-fat-diet (HFD) model of obesity, Th1 cells are abundantly present compared to control-diet- (CD) fed mice in both subcutaneous and visceral adipose tissue (SAT, VAT) (25–28). Blocking Th1 functionality by using IFNγ and T-bet knock-out mice resulted in an attenuation of adipose tissue inflammation and better glucose tolerance (25, 45, 46) (Figure 1A). This finding was confirmed by an adoptive transfer of Th1 cells to αβ T cell-deficient HFD-fed mice (28) Interestingly, T-bet knock-out mice developed a greater total body weight and VAT mass, whereas this was not the case in IFNγ knock-out mice (25, 45, 46). Notably, the metabolic alterations in T-bet-deficient mice were associated with an increased VAT expression of leptin, peroxisome proliferator receptor (PPAR) γ and CCAAT-enhancer-binding proteins (C/EBP) α (46). In contrast, Eljaafari et al. describe a decrease in IFNγ production by Th1 cells when these are co-cultured with stem cells derived from adipose tissue of obese patients (47). The discrepancy in Th1 involvement between glucose metabolism and obesity was also found in humans, as Th1 involvement has been demonstrated in DM2 patients (37–40), but not obese individuals without DM2 (Table 1) (32–35, 38, 48). However, no association with DM2 severity was observed (37–39, 48).

**Table 1:** Overview of descriptive animal and human studies concerning the presence of Th1 cells in liver, visceral adipose tissue, subcutaneous adipose tissue, and peripheral blood in NAFLD and obesity.

**Th1 Cells**					
**References**	**Study design**	**Liver**	**VAT**	**SAT**	**Blood**
**ANIMAL STUDIES**					
Rocha et al. (25)	C57BL/6 on 40% HFD for 21 wks		↑^a^		
Winer et al. (26)	C57BL/6 on 60% HFD for 8-12 wks		↑	↑	
Hong et al. (27)	C57BL/6 on 60% HFD for 10 wks		↑		
Khan et al. (28)	C57BL/6 on 21% HFD for 12 wks		↑		
Rolla et al. (29)	C57BL/6 on MCDD for 1-2-4-8 wks	↑^b^			
Kremer et al. (30)	C57BL/6 on CDD for 6 wks + ConA	↑^c^			
Ma et al. (31)	MYC-ON on MCDD for 4 wks	=			
**HUMAN STUDIES**					
Jung et al. (32)	37 overweight pediatric pts vs. 42 lean controls (aged 13–17y)				=^d^
Sumarac-Dumanovic et al. (33)	26 obese female pts vs. 20 lean age-matched female controls				=^e^
van der Weerd et al. (34)	13 obese pts (BMI > 40 kg/m^2^) vs. 25 lean controls				=
Zeyda et al. (35)	20 obese pts (BMI > 40 kg/m^2^) vs. lean-overweight controls				=^f^
Pacifico et al. (36)	50 obese pediatric pts (mean age 9.9y) vs. 20 lean controls (mean age 8.6y)				↑
Zeng et al. (37)	181 DM2 pts vs. 117 normoglycemic age-matched controls				↑
Zhao et al. (38)	90 DM2 pts vs. 30 MHO vs. 30 lean controls				↑^g^
Guo et al. (39)	31 new-onset DM2 pts vs. 16 gender and age-matched controls				↑^h^
Vonghia et al. (40)	28 NASH pts vs. 12 obese no-NASH pts				=
Rau et al. (41)	51 NASH pts vs. 30 NAFL pts vs. 43 controls				↑^i^
	18 NASH pts vs. 35 NAFL pts	=			=
Bertola et al. (42)	6 NASH pts vs. 6 S3 obese pts vs. 6 obese S0 pts	↑^j^	=		
Ferreyra et al. (43)	15 pediatric NASH pts (aged 8–15y) vs. 30 age-matched lean controls (aged 6-18y)				↑
Inzaugarat et al. (44)	20 NASH pts vs. 30 age-matched lean controls				↑

a *Assessed by CD3^+^ IFNγ^+^ cells*.

b *From 8 wks*.

c *Assessed by mRNA expression of IFNγ, TNFα, IL-12, and T-bet*.

d *Assessed by IFNγ levels*.

e *Assessed by levels of IFNγ and IL-12*.

f *Assessed by differential mRNA expression of TBX21*.

g *DM2 > MHO = controls*.

h *Although no difference was observed in serum IFNγ levels*.

i *NASH = NAFL > controls*.

j *Assessed by differential mRNA expression of IL-1β, IL-6, TNFα and IFNγ in NASH pts compared to pts with a histological grade 0 and grade 3 steatosis*.

*BMI, body mass index; CD, cluster of differentiation; ConA, concanavalin A; DM2, type 2 diabetes mellitus; HFD, high-fat diet; IFNγ, interferon γ; IL, interleukin; MCDD, methionine-choline-deficient diet; MHO, metabolically healthy obese; MYC-ON, inducible liver-specific MYC oncogene transgenic mice; NAFL, non-alcoholic fatty liver; NASH, non-alcoholic steatohepatitis; pts, patients; SAT, subcutaneous adipose tissue; Th1, T helper 1; TNFα; tumor necrosis factor α; VAT, visceral adipose tissue; wks, weeks*.

Concerning Th1 involvement in NAFLD, very little murine data exists. Rolla et al. describe an increase in liver Th1 cells in a methionine-choline-deficient-diet model of NASH (Table 1). However, this effect only occurred late in the disease progression and therefore the authors speculate that this was related to the development of fibrosis (29). Additionally, Kremer et al. describe an increase in the hepatic expression of the Th1-related cytokines IFNγ, IL-12, and TNFα after concanavalin-A-induced hepatitis in choline-deficient-diet-fed steatotic mice, which was associated with an increase in STAT4 and T-bet expression (30). Fortunately, more data are available from human studies: Rau et al. showed an increase in Th1 cells in peripheral blood of NAFLD patients compared to healthy controls (Table 1). However, no difference was found in Th1 cell numbers when comparing NAFL to NASH patients, neither in peripheral blood, nor in liver parenchyma (41). Conversely, Bertola et al. describe an upregulation of genes toward a Th1 phenotype when comparing NASH patients to patients without NASH, including NAFL patients and obese patients without liver disease (42). This finding is supported by other human studies that report an increase in peripheral blood Th1 cells in NASH patients (36, 43, 44). Moreover, Th1 cells were shown to have an antifibrotic effect, probably in an IFNγ-dependent manner (49, 50), although no fibrosis-specific data is available in the context of NAFLD.

### T Helper 2 Cells

T helper 2 (Th2) cells ensure protective immunity against parasitic infections, including helminths, and play a key role in the pathogenesis of allergic diseases (Figure 1B) (51). They express the characteristic transcription factor GATA3 and mainly produce IL-4, IL-5, and IL-13, through activation of STAT5 and STAT6, as well as the anti-inflammatory cytokine IL-10. The critical cytokines for Th2 differentiation are IL-2 and IL-4 itself, through STAT6 activation (23, 51).

In obesity-related conditions, Th2 cells seem to exert an anti-inflammatory effect, although little studies have thoroughly investigated the subset in this context. Winer et al. demonstrated a decreased number of Th2 cells in VAT of HFD-fed mice relative to other T helper subsets (Table 2) and showed that transferring CD4^+^ T cells from a population of healthy donor mice to lymphocyte deficient (RAG^null^) HFD-fed mice resulted in a decrease in total body weight, insulin resistance, and serum adipokine levels (Figure 1B). These effects were induced by differentiation of the transferred cells toward a Th2 phenotype, as assessed by *in vitro* production of IL-4 and IL-13 by T cells isolated from VAT. Additionally, transfer of CD4^+^ cells from STAT6-deficient donor mice failed to elicit the same results, confirming a Th2-dependent effect (26). Moreover, Ricardo-Gonzalez et al. demonstrated that the beneficial action of the IL-4/STAT6 axis on insulin sensitivity is dependent of inhibition of PPARα activation and attenuation of adipose tissue inflammation (52). However, it remains to be confirmed whether Th2 cells are the main source of IL-4 in this context, as the cytokine is also secreted by eosinophils and adipocytes (53, 54). In humans, there is conflicting evidence for the involvement of Th2 cells in obesity. In a gene expression study by Zeyda et al. comparing healthy obese subjects to age- and sex-matched lean or overweight controls, expression of GATA3 was differentially altered in the VAT and SAT, respectively being decreased and increased (Table 2). Furthermore, these findings corresponded to a respective increase and decrease in the TBX21/GATA3 ratio, reflecting the Th1/Th2 balance (35). Other studies present evidence for both a decrease and an increase in Th2 cells in peripheral blood of obese subjects (Table 2) (32, 34).

**Table 2:** Overview of descriptive animal and human studies concerning the presence of Th2 cells in liver, visceral adipose tissue, subcutaneous adipose tissue, and peripheral blood in NAFLD and obesity.

**Th2 Cells**					
**References**	**Study design**	**Liver**	**VAT**	**SAT**	**Blood**
**ANIMAL STUDIES**					
Winer et al. (26)	C57BL/6 on 60% HFD for 8–12 wks		↓		
Ma et al. (31)	MYC-ON on MCDD for 4 wks	=			
**HUMAN STUDIES**					
Zeyda et al. (35)	20 obese pts (BMI > 40 kg/m^2^) vs. lean-overweight controls		↓^a^	↑^a^	
Jung et al. (32)	37 overweight pediatric pts vs. 42 lean controls (aged 13–17y)				↓^b^
van der Weerd et al. (34)	13 obese pts (BMI > 40 kg/m^2^) vs. 25 lean controls				↑
Rau et al. (41)	51 NASH pts vs. 30 NAFL pts vs. 43 controls				↑^c^
	18 NASH pts vs. 35 NAFL pts	=			=
Ferreyra et al. (43)	15 pediatric NASH pts (aged 8–15y) vs. 30 age-matched lean controls (aged 6-18y)				=
Inzaugarat et al. (44)	20 NASH pts vs. 30 age-matched lean controls				=

a *Assessed by differential mRNA expression of GATA3*.

b *Assessed by decreased IL-4 levels*.

c *NASH = NAFL > controls*.

*BMI, body mass index; HFD; high-fat diet; IL, interleukin; MCDD, methionine-choline-deficient diet; MYC-ON, inducible liver-specific MYC oncogene transgenic mice; NAFL, non-alcoholic fatty liver; NASH, non-alcoholic steatohepatitis; pts, patients; SAT, subcutaneous adipose tissue; Th2, T helper 2; VAT, visceral adipose tissue; wks, weeks*.

Regarding the role of the Th2 subset in NAFLD, data are scarce as well. Rau et al. describe an increase in peripheral blood Th2 cells of NAFLD patients compared to healthy normal-weight controls, who were unfortunately not matched for age (Table 2). Moreover, they report an increase in the Th2/Treg ratio, which was subsequently shown to be decreased 12 months after bariatric surgery. However, no differences were observed between NASH patients and NAFL patients, neither in peripheral blood, nor in the liver (41). This finding is supported by other authors, who were unable to find any differences in systemic Th2 numbers between NASH patients and controls (43, 44). To our knowledge, the involvement of the Th2 subset has not been thoroughly investigated in an animal model of NAFLD. Interestingly, Th2 cells are considered to have a highly profibrogenic potential, especially through the action of IL-13, both in a tumor necrosis factor (TGF) β-dependent and -independent manner, although, to our knowledge, this aspect has not been studied in the context of NAFLD (49, 50).

### T Helper 17 Cells

T helper 17 (Th17) cells are highly proinflammatory cells that express the characteristic transcription factor retinoic acid receptor-related orphan receptor γt (RORγt), as well as STAT3 (Figure 1C). Their main action is to stimulate inflammatory processes and reinforce the adaptive cellular immune response against extracellular bacteria, fungi, and viruses through secretion of the archetypal cytokine IL-17, specifically the isoforms IL-17A and IL-17F, as well as IL-22 and IL-23. As the IL-17 receptor is expressed ubiquitously on epithelial cells, endothelial cells, monocytes, and macrophages, IL-17 brings about a powerful proinflammatory response by stimulating secretion of proinflammatory molecules. Differentiation toward the Th17 phenotype is driven by multiple cytokines: IL-6, TGFβ, IL-21, and IL-23 through activation of STAT3, as well as IL-1β and TNFα to a lesser extent (23).

The involvement of Th17 cells in obesity and NAFLD has been investigated extensively. Both animal (26, 27, 55–58) and human data (33, 34, 37, 39, 58–63) support an increase in Th17 in adipose tissue and peripheral blood in obesity and DM2, albeit in varying degrees (Table 3). Interestingly, Bertola et al. demonstrated that this accumulation might be mediated by CD11c^high^ F4/80^low^ CX3CR1^+^ DCs: in obese mice these DCs enhanced differentiation toward the Th17 subset, whereas this was the case to a substantially smaller extent in lean mice, possibly through the induction of Treg cells mediated by the presence of CD11c^+^ CD103^+^ DCs. Notably, in humans, the authors showed that CD11c^+^ CD1c^+^ DCs correlated positively with Th17 cells, as well as with obesity and insulin resistance (58). Moreover, multiple animal and *in vitro* studies have shown that IL-17 paradoxically inhibits adipogenesis (Figure 1C), at least in part by downregulating specific proadipogenic transcription factors (27, 47, 55, 57, 67, 68), including PPARγ and C/EBPα (69). Nevertheless, Th17 cells have been shown to sustain adipose tissue inflammation by ensuring a positive feedback mechanism, stimulating IL-6 and IL-1β secretion by adipocytes, macrophages and monocytes (47, 55, 59, 68). Additionally, it has been shown that IL-17 reduces hepatic, muscle and adipose tissue insulin sensitivity (27, 47, 55, 57, 60, 67).

**Table 3:** Overview of descriptive animal and human studies concerning the presence of Th17 cells in liver, visceral adipose tissue, subcutaneous adipose tissue, and peripheral blood in NAFLD and obesity.

**Th17 Cells**					
**References**	**Study design**	**Liver**	**VAT**	**SAT**	**Blood**
**ANIMAL STUDIES**					
Gomes et al. (55)	C57BL/6 on 45% HFD for 4 wks				↑
Hong et al. (27)	C57BL/6 on 60% HFD for 10 wks		↑		
Winer et al. (26)	C57BL/6 on 60% HFD for 8–12 wks		=	↑	
Zúñiga et al. (57)	C57BL/6 on 60% HFD for 36 wks		=	↑	
Bertola et al. (58)	C57BL/6 on 45% HFD for 15 wks		↑		
	10-12 wks-old db/db on standard chow		↑		
Vonghia et al. (56)	C57BL/6 on 60% HFD for 36 wks	↑	↑	=	=
Rolla et al. (29)	C57BL/6 on MCDD for 1-2-4-8 wks	↑			
Tang et al. (64)	C57BL/6 on 59% HFD for 8 wks	↑			
Giles et al. (65)	C57BL/6 on MCDD for 4 wks	↑			
He et al. (66)	C57BL/6 on 15% HFD for 16–24 wks	↑^a^			↑^b^
Ma et al. (31)	MYC-ON on MCDD for 4 wks	↑			
**HUMAN STUDIES**					
Fabbrini et al. (60)	13 MAO pts vs. 12 MHO pts vs. 9 lean controls^c^				↑^d^
Sumarac-Dumanovic et al. (33)	26 obese women vs. 20 lean controls				↑^e^
van der Weerd et al. (34)	13 obese pts (BMI > 40 kg/m^2^) vs. 25 lean controls				↑
Gomes et al. (55)	17 obese and non-obese pts	↑^f^			
Bertola et al. (58)	12-14 obese pts vs. 6-7 overweight pts vs. 10–12 lean controls			↑^g^	
Roohi et al. (62)	38 DM2 pts vs. 40 controls				=^e^
Zeng et al. (37)	181 DM2 pts vs. 117 normoglycemic age-matched controls				↑
Zhao et al. (63)	90 DM2 pts vs. 30 MHO vs. 30 lean controls				↑^h^
Guo et al. (39)	31 new-onset DM2 pts vs. 16 gender and age-matched controls				↑^i^
Dalmas et al. (59)	10 DM2 pts vs. 13 MHO pts vs. 5 controls		↑^j^		
Jagannathan-Bogdan et al. (61)	10 DM2 pts vs. 11 non-diabetic controls				↑
Vonghia et al. (40)	28 NASH pts vs. 12 obese no-NASH pts				=^b^
Rau et al. (41)	18 NASH pts vs 35 NAFL pts	↑			=^k^
	51 NASH pts vs. 30 NAFL pts vs. 43 controls				=
Tang et al. (64)	14 NASH pts vs. 4 controls	↑^l^			

a *Assessed by mRNA expression of RORγt, from 16 wks*.

b *Assessed by increased IL-17 levels*.

c *Glucose metabolism was assessed through a hyperinsulinemic-euglycemic clamp procedure. Metabolically abnormal pts were defined as subjects in the lowest tertile (glucose infusion rate) and metabolically normal pts were defined as subjects in the highest tertile (glucose infusion rate)*.

d *MAO > MHO = controls*.

e *Assessed by increased levels of IL-17 and IL-23*.

f *Positive correlation between IL-17-producing cells and degree of steatosis*.

g *obese = overweight > lean*.

h *Concerning Th17 cells: DM2 = MHO > controls, concerning IL-17: DM2 > MHO = controls*.

i *Although no difference was observed in IL-17 levels*.

j *DM2 > MHO > controls*.

k *Although an increase in the Th17/rTreg ratio was observed*.

l *Assessed by mRNA expression of RORγt, IL-17, IL-21, and IL-23*.

*BMI, body mass index; DM2, type 2 diabetes mellitus; HFD; high-fat diet; IL, interleukin; MAO, metabolically abnormal obese; MCDD, methionine-choline-deficient diet; MHO, metabolically healthy obese; MYC-ON, inducible liver-specific MYC oncogene transgenic mice; NAFL, non-alcoholic fatty liver; NASH, non-alcoholic steatohepatitis; pts, patients; RORγt, RAR-related orphan receptor γt; rTreg, resting regulatory T cells; SAT, subcutaneous adipose tissue; Th17, T helper 17; VAT, visceral adipose tissue; wks, weeks*.

Th17 cells were shown to be present in larger numbers in the liver and peripheral blood in animal models of NAFLD (Table 3) (29, 31, 56, 64–66). The same is true for human NAFLD, particularly when comparing patients with NASH to patients without NASH (40, 41, 55, 64). Moreover, Rau et al. report a decrease in peripheral blood Th17 cells, as well as in the Th17/Treg ratio, when NASH patients were re-evaluated 12 months after bariatric surgery (41). However, data about the exact role of Th17 cells in the development of steatosis is equivocal (Figure 1C). Multiple experimental animal and *in vitro* studies report an increase in steatosis when administering IL-17, as well as a decrease in steatosis when blocking IL-17 functionality (29, 55, 64, 70). In contrast to the situation in adipose tissue, IL-17 has been shown to increase the hepatic expression of PPARγ (55), while blocking IL-17 functionality did not induce differences in the hepatic expression of PPARα or sterol regulatory element-binding protein (SREBP) 1c, all important regulators of lipid metabolism (64, 65). Conversely, other authors report an increase in steatosis when IL-17 functionality is inhibited (65, 67). On the other hand, the detrimental effect of Th17 cells on liver inflammation (64, 65, 67, 70, 71) and liver damage, as assessed by a rise in transaminases (29, 64, 65, 67, 70) is unequivocal. This Th17-induced hepatic inflammation might result from the accumulation of macrophages through IL-17-dependent upregulation of C-X-C motif chemokine (CXCL) 10, a powerful chemoattractant (65, 70). Alternatively, Rolla et al. have shown *in vitro* that the known lipotoxic effects of fatty acids are exacerbated in the presence of IL-17 in a c-Jun N-terminal kinase (JNK)-dependent manner (29). Moreover, Tang et al. showed *in vitro* that HepG2 cells produce IL-6, induced by the synergistic action of free fatty acids and IL-17, which suggests the presence of the same positive feedback mechanism for Th17 differentiation described in adipose tissue (64). Lastly, Th17 cells have a clear fibrogenic effect, likely due to the direct action of IL-17 on hepatic stellate cells by inducing collagen production in a JNK- and STAT3-dependent manner (49, 71–75).

### T Helper 22 Cells

T helper 22 (Th22) cells are characterized by the production of IL-22 in absence of other major cytokines, most importantly IL-17 (Figure 1D) (76). Through IL-22 secretion, Th22 cells enhance the innate immunity of epithelia and play a fundamental role in the elimination of bacterial infections at body surfaces (77). Th22 cell differentiation is driven by IL-6 and TNFα and inhibited in the presence of TGFβ. The Th22 cell's production of IL-22 depends on activation of the transcription factor aryl hydrocarbon receptor (AHR) (76).

The Th22 subset and its effector cytokine IL-22 were shown to be expanded in the adipose tissue and peripheral blood of obese individuals and DM2 patients compared to controls (Table 4) (38, 39, 59, 60). Although the work of some authors might suggest that this constitutes a protective effect toward the development of obesity (Figure 1D) (78, 79), Park et al. argue that low, physiologic levels IL-22 are unlikely to contribute to the pathogenesis of HFD-induced obesity and its metabolic sequelae, which is supported by other studies (68, 80). Moreover, conflicting evidence exists in regard to whether or not IL-22 has a beneficial effect on glucose metabolism (78–81), although the same study by Park et al. reports an IL-22-induced inhibition of hepatic gluconeogenesis in a STAT3 and AMP-activated protein kinase- (AMPK) dependent manner (80).

**Table 4:** Overview of descriptive animal and human studies concerning the presence of Th22 cells in liver, visceral adipose tissue, subcutaneous adipose tissue, and peripheral blood in NAFLD and obesity.

**Th22 Cells**					
**References**	**Study design**	**Liver**	**VAT**	**SAT**	**Blood**
**ANIMAL STUDIES**					
Jung et al. (32)	C57BL/6 on 35% HFD for 15 wks				↓^a^
Rolla et al. (29)	C57BL/6 on MCDD for 1-2-4-8 wks	↑^b^			
**HUMAN STUDIES**					
Fabbrini et al. (60)	13 MAO pts vs. 12 MHO pts vs. 9 lean controls^c^			↑^d^	
Zhao et al. (38)	90 DM2 pts vs. 30 MHO vs. 30 lean controls				↑^e^
Guo et al. (39)	31 new-onset DM2 pts vs. 16 gender and age-matched controls				↑
Dalmas et al. (59)	10 DM2 pts vs. 13 MHO pts vs. 5 controls		↑^f^		

a *Assessed by decreased IL-22 levels*.

b *Only at 2 and 4 wks*.

c *Glucose metabolism was assessed through a hyperinsulinemic-euglycemic clamp procedure. Metabolically abnormal patients were defined as subjects in the lowest tertile (glucose infusion rate) and metabolically normal patients were defined as subjects in the highest tertile (glucose infusion rate)*.

d *MAO > MHO > controls, Th22 cells were defined as CD4+ IL-22-producing cells*.

e *DM2 > MHO > controls*.

f *DM2 > controls*.

*CD, cluster of differentiation; DM2, type 2 diabetes mellitus; HFD; high-fat diet; IL, interleukin; MAO, metabolically abnormal obese; MCDD, methionine-choline-deficient diet; MHO, metabolically healthy obese; pts, patients; SAT, subcutaneous adipose tissue; Th22, T helper 22; VAT, visceral adipose tissue; wks, weeks*.

In experimental settings, IL-22 seems to have a beneficial effect on multiple components of NAFLD (Figure 1D). Recombinant IL-22 was shown to alleviate steatosis and decrease transaminase levels (79, 81), possibly through a STAT3-mediated mechanism (79). Interestingly, short-term treatment induced a decrease in hepatic expression of the lipogenic proteins PPARα, PPARγ, and SREBP-1c. In contrast, long-term treatment with IL-22 did not induce this effect, but decreased the hepatic expression of fatty acid synthase (FAS), a central enzyme in lipogenesis, and “elongation of very long chain fatty acids protein 6” (ELOVL6), a protein that elongates long-chain fatty acids (81). Additionally, Rolla et al. showed that IL-22 attenuates palmitate lipotoxicity *in vitro* via phosphoinositide 3-kinase (PI3K)/Akt-dependent inhibition of JNK, which might explain the decrease in transaminase levels. Importantly, this protection was lost in the presence of IL-17, which upregulated the PI3K/Akt inhibitor “phosphatase and tensin homolog deleted on chromosome 10” (PTEN) (29). Lastly, IL-22 was shown to also have an antifibrogenic effect (72). It should be noted, however, that in addition to the beneficial effects described above, IL-22 also promotes the development of hepatocellular carcinoma in a STAT3-dependent manner (82).

### Regulatory T Cells

Regulatory T (Treg) cells are cells that express the transcription factor forkhead box P3 (Foxp3) (Figure 1E) and exert an immune-controlling effect. Their main action is to prevent autoreactivity toward self-antigens and to avoid excessive effector-T-cell activation and subsequent tissue damage during infection-induced immune responses (23, 51). Treg cells exert this function by the production of the inhibitory cytokines IL-10 and TGFβ, by interference with T-cell survival through IL-2 depletion and by secretion of molecules that directly eliminate effector cells and inhibit antigen-presenting-cell maturation and functionality. Natural Treg cells originate in the thymus and form a relatively stable compartment, whereas induced Treg cells differentiate out of naïve T cells in the periphery when the appropriate stimuli are present (83). Treg-cell differentiation is driven by TGFβ in the absence of IL-6 and is further reinforced by IL-2 and retinoic acid through STAT5 activation (23). Indeed, in the differentiation of Treg and Th17 cells, an important role is reserved for IL-6, as it determines the direction of the differentiation pathway, i.e., toward the Treg subset in its absence and toward the Th17 subset in its presence. Moreover, Foxp3 can directly inhibit Th17 cell differentiation through direct interaction with RORγt, further influencing the balance between the two subsets. Additionally, a large degree of plasticity has been demonstrated in Treg cells, as they may differentiate into Th17 cells when the appropriate stimuli are present. Therefore, the Treg/Th17 balance has been used as a model to study the involvement of the subsets in multiple disease states (23).

Feuerer et al. demonstrated that Treg cells are highly enriched in VAT, but not in SAT, of lean mice and that they possess a transcriptionally unique phenotype compared to Treg cells in peripheral lymphoid tissues (84). In animal models of obesity, decreased levels of Treg cells have been demonstrated in the spleen and VAT (26, 84, 85), while Vonghia et al. report an increase in relative Treg cell numbers in SAT, which might indicate a differential role for SAT in obesity (Table 5) (56). Interestingly, a negative correlation was found between VAT Treg cell numbers and insulin resistance (84) and multiple experimental gain-of-function and loss-of-function studies have confirmed an attenuating effect of VAT Treg cells on insulin resistance (Figure 1E) (84, 93, 94). Compellingly, Cipolletta et al. have shown that Treg functionality in VAT is PPARγ dependent (93). The investigators created a transgenic mouse line in which PPARγ is specifically deleted in Treg cells and showed that, in these mice, the PPARγ agonist pioglitazone loses its beneficial effect on glucose tolerance. Of note, this beneficial effect was present in WT littermates and was associated to increases in Treg cell numbers in VAT (93), as well as in the liver (85). Conversely, TGFβ, known to be secreted by Treg cells, was shown to be associated with adiposity and its neutralization decreased body weight and insulin resistance (23, 95). In humans, some discrepancy exists between the available studies (Table 5): analogous to the situation in mice, several studies in obese and diabetic patients show a reduction in VAT and peripheral blood Treg cells (61, 89–91), whereas other authors describe a paradoxical increase in both the VAT and SAT of obese individuals (35, 87, 88, 96).

**Table 5:** Overview of descriptive animal and human studies concerning the presence of Treg cells in liver, visceral adipose tissue, subcutaneous adipose tissue, and peripheral blood in NAFLD and obesity.

**Treg Cells**					
**References**	**Study design**	**Liver**	**VAT**	**SAT**	**Blood**
**ANIMAL STUDIES**					
Feuerer et al. (84)	C57BL/6 on 60% HFD for 29 wks		↓		
Xu et al. (85)	C57BL/6 on 15% HFD for 10 wks		↓		
Winer et al. (26)	C57BL/6 on 60% HFD for 8-12 wks		↓	=	
Vonghia et al. (56)	C57BL/6 on 60% HFD for 36 wks	=	=	↑	=
Ma et al. (86)	C57BL/6 on 50% HFD for 1-2-4-8 wks	↓			
He et al. (66)	C57BL/6 on 15% HFD for 8-16–24 wks	↓^a^			
Ma et al. (31)	MYC-ON on MCDD for 4 wks	↓^b^			
**HUMAN STUDIES**					
Donninelli et al. (87)	15 obese pts vs. 16 lean controls		↑		=
Pereira et al. (88)	16 obese pts (BMI > 40 kg/m^2^) vs. 15 sex- and age-matched lean controls		=^c^	↑^c^	↑^d^
van der Weerd et al. (34)	13 obese pts (BMI > 40 kg/m^2^) vs. 25 lean controls				↑
Zeyda et al. (35)	20 obese pts (BMI > 40 kg/m^2^) vs. lean-overweight controls		↑	↑	
Deiuliis et al. (89)	6 obese pts vs. 6 lean controls		↓^e^		
Winer et al. (26)	Obese pts vs. lean controls		↓^f^		
Wagner et al. (90)	30 obese (BMI > 27 kg/m^2^) vs. normal-weight controls (BMI < 27 kg/m^2^)				↓
Esser et al. (91)	19 MAO pts vs. 15 MHO pts vs. 7 lean controls		↓^g^	=	
Zeng et al. (37)	181 DM2 pts vs. 117 normoglycemic age-matched controls				↓
Jagannathan-Bogdan et al. (61)	10 DM2 pts vs. 11 non-diabetic controls				↓
Vonghia et al. (40)	28 NASH pts vs. 12 obese no-NASH pts				↓^h^
Söderberg et al. (92)	33 NASH pts (NAS 3-6) vs. 12 no-NASH pts (NAS 0-2)	↑^i^			
Rau et al. (41)	18 NASH pts vs. 35 NAFL pts	↓^j^			
	30 NASH pts vs. 51 NAFL pts vs. 43 controls				↓^k^
Bertola et al. (42)	6 NASH pts vs. 6 S3 obese pts vs. 6 obese S0 pts	↑^l^			

a *Assessed by mRNA expression of Foxp3, only at 16 wks*.

b *CD4^+^ Foxp3^+^ cells, only in absolute cell numbers*.

c *Assessed by mRNA expression of IL-10, TGFβ and Foxp3*.

d *Assessed by increased IL-10 levels*.

e *Assessed by mRNA expression of Foxp3*.

f *Assessed immunohistochemically by an increased T-bet/Foxp3 ratio*.

g *MAO > MHO = control patients*.

h *Assessed by a decreased IL-10/IL-17 ratio*.

i *Assessed by immunohistochemical staining of Foxp3*.

j *Assessed by an increased Th17/rTreg ratio*.

k *Specifically, rTreg cells were assessed: NASH < NAFL < controls*.

l *Assessed by an increased IL-10/IFNγ ratio*.

*BMI, body mass index; DM2, type 2 diabetes mellitus; Foxp3, forkhead box P3; HFD; high-fat diet; IFNγ, interferon γ; IL, interleukin; iNKT, invariant natural killer T; MAO, metabolically abnormal obese; MCDD, methionine-choline-deficient diet; MHO, metabolically healthy obese; MYC-ON, inducible liver-specific MYC oncogene transgenic mice; NAFL, non-alcoholic fatty liver; NAS, NAFLD activity score; NASH, non-alcoholic steatohepatitis; pts, patients; rTreg; resting Treg cells; SAT, subcutaneous adipose tissue; Th17, T helper 17; Treg, regulatory T; VAT, visceral adipose tissue; wks, weeks*.

Although data are scarce, hepatic Treg cell numbers are reduced in animal models of NAFLD (Table 5) (31, 66, 86, 97). According to Ma et al., this reduction is due to local reactive-oxygen-species- (ROS) induced apoptosis of Treg cells, which could be reversed by concurrent treatment with the antioxidant MnTBAP (86). Moreover, the authors showed that an adoptive transfer of Treg cells could attenuate HFD-induced hepatic inflammation, as assessed by a decrease in hepatic TNFα expression (Figure 1E) (86). Ilan et al. showed that steatosis and elevated transaminase levels could be attenuated in a leptin-deficient murine model by expanding Treg cell numbers through oral administration of anti-CD3 antibodies and β-glucosylceramide. However, the authors do not report whether this actually had an effect on hepatic Treg cell numbers, although they did demonstrate an increase in VAT Treg cell numbers (94). As is the case in obesity, the opposite is observed in human liver steatosis, where most available studies suggest an increase in liver Treg cell numbers, albeit via indirect characterization of the subset (Table 5) (41, 42, 92). Although, again, no studies are available in the context of NAFLD, most evidence suggests an antifibrotic effect of Treg cells, through the secretion of IL-10 (98). In contrast, TGF-β has been shown to be involved in the development of both hepatic steatosis and fibrosis (23, 95, 99, 100). This could imply a dual role for this subset, although Treg cells are far from the only source of the above-mentioned cytokines.

## CD8^+^ Cytotoxic T Cells

Tc cells are the main effector cells of the cellular immune system (Figure 1F). Through recognition of antigens presented on the MHC I of virtually every possible cell type, they induce cytotoxic processes aimed at eliminating infected or malignantly transformed cells. These effects are brought about by cytokine secretion, release of cytotoxic agents—including perforin and granzymes—and direct cell-cell contact (101). Tc cells are formed in the bone marrow and mature in the thymus through subsequent positive and negative selection depending on MHC I affinity (21).

Although it has not yet been confirmed in humans, HFD animal models support an increase of Tc cells in the VAT in obesity (Table 6) (102–104). Importantly, this infiltration occurs at a very early stage, even preceding macrophage infiltration, and is dependent of the trafficking marker CD11a. Moreover, Nishimura et al. have shown that Tc cells are essential for macrophage recruitment and adipose tissue inflammation through production of chemotactic molecules, emphasizing a crucial role for the Tc cell—adipocyte interaction. Additionally, the authors demonstrated an improvement in glucose tolerance and insulin resistance when blocking Tc cell functionality, although an effect on body weight was not observed (Figure 1F). Interestingly, these detrimental effects of Tc cells were demonstrated by using neutralizing anti-CD8 antibodies in both a preventive and curative approach (102). These findings were confirmed by Ghazarian et al. in a CD8 knock-out model and by selective adoptive transfer of Tc cells. Furthermore, in humans, these authors report a positive correlation between glycated hemoglobin levels and intrahepatic Tc cells in humans (106). Conversely, Lynch et al. demonstrated decreased amounts of Tc cells in the peripheral blood of obese patients compared to lean controls (107).

**Table 6:** Overview of descriptive animal and human studies concerning the presence of Tc cells in liver, visceral adipose tissue, subcutaneous adipose tissue, and peripheral blood in NAFLD and obesity.

**Tc Cells**					
**References**	**Study design**	**Liver**	**VAT**	**SAT**	**Blood**
**ANIMAL STUDIES**					
Nishimura et al. (102)	C57BL/6 on 60% HFD for 0–26 wks		↑		
Rausch et al. (103)	C57BL/6 on 35% HFD for 12 wks		↑		
Kawanishi et al. (104)	C57BL/6 on 60% HFD for 16 wks		↑		
Bhattacharjee et al. (105)	C57BL/6 on 58% HFD for 18 wks	↑			
Ghazarian et al. (106)	C57BL/6 on 60% HFD for 10-16-32 wks	↑^a^			
**HUMAN STUDIES**					
Zeyda et al. (35)	20 obese pts (BMI > 40 kg/m^2^) vs. lean-overweight controls		=	=	
van der Weerd et al. (34)	13 obese pts (BMI > 40 kg/m^2^) vs. 25 lean controls				=
Lynch et al. (107)	26 MAO pts vs. 26 age-, BMI- and gender-matched MHO pts vs. 11 lean controls				↓^b^
Inzaugarat et al. (44)	20 NASH pts vs. 30 age-matched lean controls				↑^c^
Ferreyra et al. (43)	15 pediatric NASH pts (aged 8-15y) vs. 30 age-matched lean controls (aged 6-18y)				↑^c^
Gadd et al. (108)	13 fibrotic NASH pts (≥F2) vs. 9 non-fibrotic NASH pts vs. 11 NAFL pts vs. 10 lean controls	↑^d^			

a *From 16 weeks*.

b *MAO < MHO < lean controls*.

c *Specifically IFNγ-producing Tc cells*.

d *Assessed by immunohistochemical staining. At the portal tract more CD8^+^ cells were present in fibrotic NASH patients compared to non-fibrotic NASH patients, NAFL patients and controls*.

*BMI, body mass index; HFD; high-fat diet; IFNγ, interferon γ; NAFL, non-alcoholic fatty liver; NASH, non-alcoholic steatohepatitis; pts, patients; SAT, subcutaneous adipose tissue; Tc, cytotoxic T; VAT, visceral adipose tissue; wks, weeks*.

Some evidence exists from animal models as well as human studies for an increase in Tc cell numbers in NAFLD, albeit rather limited (Table 6) (105, 106, 108). Blocking Tc cell functionality through administration of neutralizing anti-CD8 antibodies was shown to result in a decrease in liver steatosis, liver inflammation and transaminase levels (97, 105), as well as reduced hepatic-stellate-cell activation, albeit to a minor extent (Figure 1F) (97). Interestingly, Wolf et al. underline that Tc cells alone might not suffice to elicit the observed liver damage and that concomitant NKT cell involvement is pivotal. Additionally, the authors demonstrated that both Tc cells and NKT cells are involved in NASH-to-HCC progression (97). Conversely, Ma et al. describe a ROS-dependent loss of hepatic CD4^+^ T helper cells in a combined HCC and NAFLD mouse model, which was associated to obesity-related lipid dysregulation and lead to increased hepatocarcinogenesis, whereas CD8^+^ Tc numbers were unaffected. It should be noted that further characterization of the involved Th subsets was not performed in this study (31).

## Innate T Cells

### Natural Killer T Cells

NKT cells are lymphocytes originally defined as cells expressing both the characteristic T-cell marker CD3 and natural killer cell markers (Figure 1G). Unlike conventional T cells, which require peptide presentation via the MHC, NKT cells exclusively recognize lipid antigens—mainly glycolipids and glycerol lipids—presented by the MHC-like molecule CD1d. Because some CD1d-restricted NKT cells do not express NK cell markers and activated conventional T cells can also express NK cell markers, NKT cells are currently defined as all CD1d-restricted cells and the original definition should no longer be used (109, 110). NKT cells are usually subclassified into two subtypes: invariant NKT cells (iNKT; type 1), which express a semi-invariant TCRα chain (Vα14Jα18 in mice, Vα24Jα18 in humans) paired with a very limited array of TCRβ proteins and are by far the largest subset (at least in mice); and non-invariant NKT cells (type 2), which possess a more diverse TCR repertoire. Physiologically, NKT cells act as an evolutionary bridge between the innate and adaptive immune systems, rapidly responding with the secretion of large amounts of cytokines upon antigen recognition, including IFNγ, IL-4, IL-13, and IL-17, depending upon the tissue milieu, antigen-presenting cell and lipid antigen. Importantly, NKT cells seem to be implicated in murine immunity to a substantially larger degree than in humans (up to 10 fold), comprising 0.5% of the T-cell population in peripheral blood and up to 30% in the liver sinusoids in mice (110–112).

Lynch et al. have shown, both in mice and humans, that NKT cells accumulate in adipose tissue (113, 114). Moreover, they describe a reversible decrease in NKT cell numbers in obesity in favor of proinflammatory macrophages (113), which has been confirmed by Ji et al. (Table 7) (115). Conversely, Mantell et al. report a small increase in NKT cell numbers in the adipose tissue of HFD-fed mice (120). Although multiple gain-of-function and loss-of-function experiments have been performed, the exact role of NKT cells in adiposity and glucose metabolism is yet to be clarified (Figure 1G). There is some evidence suggesting an attenuating role for NKT cells on adiposity, adipose tissue inflammation and insulin resistance (113, 115, 126–129), although this has been contradicted by at least as many studies finding no effect at all (115, 119, 120, 126, 129–131), and even some reporting an exacerbating effect (105, 119). Ji et al. suggest that the NKT cells' potentially beneficial effects on the glucose metabolism are mediated by the IL-4/STAT6 axis (115).

**Table 7:** Overview of descriptive animal and human studies concerning the presence of NKT cells in liver, visceral adipose tissue, subcutaneous adipose tissue, and peripheral blood in NAFLD and obesity.

**NKT Cells**					
**Reference**	**Study design**	**Liver**	**VAT**	**SAT**	**Blood**
**ANIMAL STUDIES**					
Ji et al. (115)	C57BL/6 on 60% HFD for 1-8-24 wks		↓^a^		
Guebre-Xabier et al. (116)	10–12 wks-old ob/ob C57BL/6 on standard chow	↓			
Li et al. (117)	C57BL/6 on 59% HFD for 4–12 wks	↓			
Yang et al. (118)	10-12 wks-old ob/ob C57BL/6 on standard chow	↓			
Wu et al. (119)	C57BL/6 on 60% HFD for 0–12 wks	↓^a^	=^a^		
Bhattacharjee et al. (105)	C57BL/6 on 58% HFD for 18 wks	↑			
Mantell et al. (120)	C57BL/6 on 44% HFD for 26 wks	↓	↑		
Miyazaki et al. (121)	C57BL/6 on 21% HFD for 3 wks	↓			
Lynch et al. (113)	C57BL/6 on 60% HFD for 6 wks	↓^a^	↓^a^		
Ghazarian et al. (106)	C57BL/6 on 60% HFD for 16 wks	↓			
**HUMAN STUDIES**					
Adler et al. (122)	6 obese pts with S2-3 vs. 11 obese pts with S1 vs. 10 obese pts with S0	↑^b^			↑^b^
Lynch et al. (114)	15 obese pts vs. 6 lean controls		↓^a^		
Ji et al. (115)	14 overweight-obese female pts vs. 25 lean female controls		↓^c^		
Kremer et al. (123)	3 S2-3 pts vs. 4 S1 pts vs. 5 S0 pts	↓^d^			
Tajiri et al. (124)	28 NASH pts (NAS ≥ 5) vs. 34 NAFL pts (NAS ≤ 4)	↑			
Xu et al. (125)	60 NAFLD pts vs. 60 lean controls				↓^a^

a *Specifically iNKT cells*.

b *S2-3 > S1 = S0*.

c *Specifically iNKT cells, assessed by Vα24 mRNA expression*.

d *S2-3 < S1 < S0*.

*HFD, high-fat diet; iNKT, invariant natural killer T; NAFL, non-alcoholic fatty liver; NAFLD, non-alcoholic fatty liver disease; NAS, NAFLD activity score; NASH, non-alcoholic steatohepatitis; NKT, natural killer T; pts, patients; SAT, subcutaneous adipose tissue; VAT, visceral adipose tissue; wks, weeks*.

Concerning NAFLD, it is now widely accepted that NKT cells are depleted in experimental, murine liver steatosis (Table 7) (113, 116–121). Interestingly, Tang et al. showed that the NAFLD-associated depletion of liver NKT cells occurs by induction of apoptosis through activation of the membrane receptor “T cell Ig and mucin domain 3” (Tim-3), a protein expressed on terminally differentiated T cells. Moreover, galectin-9, the natural ligand to Tim-3, was shown to elicit a paradoxical proliferation of Tim-3^−^ NKT cells in an IL-15- and Kupffer-cell-dependent manner, as well as an attenuation of HFD-induced steatosis and total body weight in mice (127). Additionally, Kremer et al. have shown that the observed NKT-cell depletion is dependent of IL-12 secreted by Kupffer cells (123). Multiple other studies confirm that NKT cells might have an attenuating effect on various aspects of NAFLD, attenuating steatosis (113, 128, 129), as well as diminishing liver inflammation and transaminase levels (Figure 1G) (130). Again, these findings have been contradicted by other studies, reporting no or the reverse effect (97, 105, 119, 120, 129, 130). Kotas et al. argue that, although the observed beneficial effects are CD1d mediated, they are not iNKT dependent, as deletion of the iNKT-specific Jα18 segment of the semi-invariant TCR failed to elicit the same outcome (129). Considering the effect of NKT cells on fibrosis, the evidence is contrasting as well: both Syn et al. and Wolf et al. report a fibrogenic effect through osteopontin- and sonic-hedgehog-mediated activation of hepatic stellate cells (97, 132). Conversely, Miyagi et al. demonstrated increased liver fibrosis in HFD-fed NKT-cell-deficient mice (130). The contrasting effects described above might be caused by the dichotomy between type 1 (invariant) and type 2 (non-invariant) NKT cells, which is not consequently taken into account in all studies. As is the case in mouse models, the involvement of NKT cells in human NAFLD is unclear with evidence for both an increase and decrease in liver NKT cell numbers (122–125).

### γδ T Cells

Interestingly, some studies suggest that the great majority of IL-17-producing cells in both adipose tissue and the liver are not classic Th17 cells, but IL-17-producing γδ T cells (57, 133), which make up 3–5% of all hepatic lymphocytes. These cells differ from classic αβ T cells in the composition of their TCR and, like NKT cells, constitute a component of the innate rather than the adaptive immune system. Due to this alternatively composed TCR, γδ T cells do not require MHC-dependent peptide presentation and recognize non-peptide bacterial antigens and damage-associated ligands, thus acting as a rapid responder to infections through secretion of IL-17, as well as IFNγ, depending on the specific subset (134).

Zúñiga et al. demonstrated that the numbers of IL-17-producing γδ T cells were decreased in the VAT of HFD-fed mice compared to CD-fed mice, whereas no difference was observed in conventional Th17 numbers (Table 8). Conversely, the numbers of both IL-17-producing γδ T cells and Th17 cells were shown to be increased in SAT (57).

**Table 8:** Overview of descriptive animal studies concerning the presence of γδ T cells in liver, visceral adipose tissue, subcutaneous adipose tissue, and peripheral blood in NAFLD and obesity.

****γδ**** **T Cells**					
**Reference**	**Study design**	**Liver**	**VAT**	**SAT**	**Blood**
**ANIMAL STUDIES**					
Zúñiga et al. (57)	C57BL/6 on 60% HFD for 12 wks		↓	↑^a^	
Li et al. (133)	C57BL/6 on 60% HFD for 24 wks	↑			
Ghazarian et al. (106)	C57BL/6 on 60% HFD for 16 wks	↑^b^			

a *Specifically IL-17-producing γδ T cells*.

b *Only in absolute cell numbers*.

*HFD, high-fat diet; IL, interleukin; pts, patients; SAT, subcutaneous adipose tissue; VAT, visceral adipose tissue; wks, weeks*.

Li et al. report an increase in both adipose tissue and hepatic γδ T cells, specifically IL-17-producing γδ T cells, in HFD-induced obesity and NAFLD in mice (Table 8). Moreover, in contrast to the above-mentioned studies, the authors fail to find an increase in classic Th17 cells in the liver. Furthermore, hepatic inflammation, transaminase levels, and insulin resistance were shown to be reduced when using γδ T cell-deficient *tcrd*^−/−^ mice. Additionally, the same effects were observed when γδ T-cell accumulation was inhibited by preventing antigen-mediated activation through antibiotic-induced depletion of commensals in the gut. Importantly, these effects could be reversed by administration of IL-17 (133). Because all these effects are IL-17 dependent in both γδ T cells and Th17 cells, the exact significance of these findings is not yet clear in the context of long-term metabolic diseases.

### Mucosal-Associated Invariant T Cells

Another type of T cell belonging to the innate rather than the adaptive immune system is the MAIT cell. These cells express CD161 and a semi-invariant TCR, composed of an invariant α chain, (Vα7.2Jα33 in humans and Vα19Jα33 in mice) and a limited repertoire of β chains (mostly Vβ6 and Vβ20) (135, 136). Through this TCR, MAIT cells recognize pathogen-related metabolites of vitamin B, presented on MHC class I-like molecules, called MR1, and subsequently secrete the proinflammatory cytokines TNFα, IFNγ and IL-17, as well as the cytotoxic agents granzyme B and perforin. MAIT cells are highly prevalent in humans, reaching up to 10% of T cells in the peripheral blood and the gut mucosa, and 10 to 40% of T cells in the liver (136). Interestingly, the maturation of MAIT cells was shown to be dependent of the presence of commensal flora, as they were not present in germ-free mice (137).

The possible involvement of MAIT cells in obesity and DM2 was investigated by Magalhaes et al. The authors showed that, although MAIT cell levels were reduced in the peripheral blood of obese and DM2 patients and unaltered in VAT and SAT, these MAIT cells exhibited a more proinflammatory phenotype, readily secreting IL-17, as well as increased activation and recruitment markers. Interestingly, 12 months after bariatric surgery, the peripheral blood MAIT cells reached frequencies comparable to those in lean patients, whereas they retained the IL-17-secreting phenotype (138). These data are supported by other studies by Touch et al. and Carolan et al. the latter even demonstrating a decrease in VAT MAIT cells, as well as a propensity for IL-17 production in favor of IL-10 production (Table 9) (139, 140). Although no studies are available in the context of NAFLD and further exploration of the role of MAIT cells in this disease is certainly warranted, Hegde et al. have recently shown a profibrogenic effect of MAIT cells in the liver (141).

**Table 9:** Overview of descriptive human studies concerning the presence of MAIT cells in liver, visceral adipose tissue, subcutaneous adipose tissue, and peripheral blood in NAFLD and obesity.

**MAIT Cells**					
**Reference**	**Study design**	**Liver**	**VAT**	**SAT**	**Blood**
**HUMAN STUDIES**					
Magalhaes et al. (138)	37 obese DM2 pts vs. 52 obese non-DM2 pts vs. 10 non-obese DM2 pts vs. 23 non-obese non-DM2 controls				↓^a^
	31 obese pts vs. 7 non-obese controls		=		
	7 obese pts vs. 4 non-obese controls			=	
Carolan et al. (139)	30 obese pts. vs. 35 non-obese controls				↓
	10 obese pts. vs. 8 non-obese controls		↑		
Touch et al. (140)	89 non-DM2 obese pts vs. 138 DM2 pts. vs. 43 metabolic syndrome pts. vs. 66 lean controls				↓^b^

a *Obese DM2 = obese non-DM2 = non-obese DM2 < controls*.

b *Non-DM2 obese = DM2 = metabolic syndrome < controls*.

*DM2, type 2 diabetes mellitus; MAIT, mucosal-associated invariant T; SAT, subcutaneous adipose tissue; VAT, visceral adipose tissue*.

## Interplay Between Adipose Tissue, Liver, and Gut

**Figure 2:**
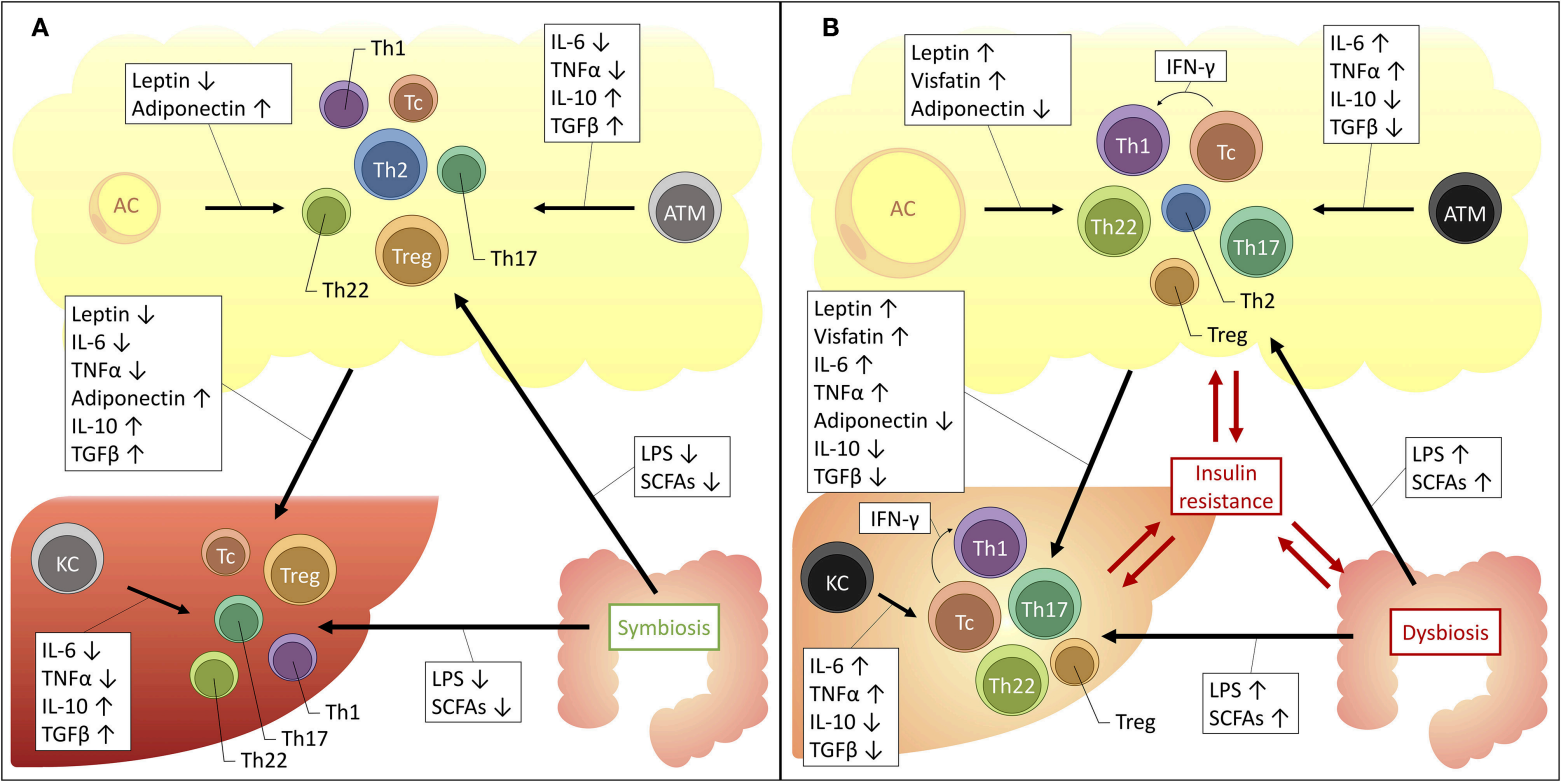
Proposed mechanisms for the interplay between the liver, adipose tissue and gut microbiome in obesity and NAFLD. The increased availability of a substance is depicted by an upwards arrow, the decreased availability by a downwards arrow. **(A)** Situation in health. **(B)** Situation in obesity and NAFLD. AC, adipocyte; ATM, adipose tissue macrophage; IFNγ, interferon γ; IL, interleukin; KC, Kupffer cell; LPS, lipopolysaccharide; SCFAs, short-chain fatty acids; TGFβ, transforming growth factor β; Tc, cytotoxic T cell; Th1, T helper 1 cell; Th17, T helper 17 cell; Th2, T helper 2 cell; Th22, T helper 22 cell; TNFα, tumor necrosis factor α; Treg, regulatory T cell.

From the above, it should be clear that distinct T-cell subsets are involved in the adipose tissue and hepatic inflammation associated with obesity, insulin resistance and NAFLD. As described above, these conditions are intimately linked, both clinically and pathophysiologically. Consequently, a relationship between the hepatic and adipose tissue T-cell profile could be expected. Indeed—although for the most part they remain to be formally confirmed—several driving mechanisms could be proposed (Figure 2).

Firstly, an important role is reserved for macrophages. These cells of the innate immune system are abundantly present in both the adipose tissue and the liver and were shown to be critically involved in the pathophysiology of both obesity and NAFLD (20, 142). At a local level, in both disease states, it has been established that macrophages take on a more proinflammatory phenotype, characterized by the secretion of proinflammatory cytokines, including IL-6, TNFα, IL-1β, IL-12, and IL-23 (142, 143). These cytokines are well-established drivers of T-cell differentiation toward the Th1, Th17, and Th22 subsets, while inhibiting differentiation toward the Th2 and Treg subsets (23). Moreover, a direct link between ATMs and liver macrophages has already been established (144), certainly making it conceivable that the same might be true for liver T cells, although this remains to be formally proven.

Secondly, one should consider the important regulatory effect that the adipose tissue exerts systemically, including with respect to the liver. Since the discovery of leptin in 1994, it has been accepted that the adipose tissue is much more than a mere site for excess energy storage and that it serves as a fully capable endocrine organ, regulating satiety, glucose metabolism, and fatty acid oxidation. The key regulators in this context are adipokines, collectively encompassing hormones and cytokines secreted by the adipose tissue, either by adipocytes or cells populating the stromal vascular fraction, including the above-mentioned ATMs. Importantly, the composition of the secreted adipokines changes in the context of obesity: in lean persons the adipose tissue predominantly secretes anti-inflammatory adipokines, including adiponectin, TGF-β, IL-4, IL-13, and IL-10. However, in case of obesity the secreted adipokines take on a more proinflammatory profile, secreting IL-6, TNFα and leptin, as well as resistin and visfatin. Leptin, a hormone that induces satiety and increased energy expenditure was shown to be paradoxically increased in obesity, leading to the use of the concept of leptin resistance. Moreover, IL-6 and TNFα levels have both been found to be increased in the serum of obese patients and are critical drivers of obesity-associated insulin resistance (145). Interestingly, many of these adipokines exhibit regulatory functions over T-cell differentiation, inducing anti-inflammatory T-cell subsets in physiologic conditions and proinflammatory T-cell subsets in the context of obesity. Indeed, as outlined before, the role of IL-6 and TNFα as key regulators of the Th17/Th22/Treg equilibrium is already well-established (23, 76). However, as depicted in Table 10, multiple other–more adipose tissue-specific—adipokines can also induce distinct T-cell subsets. Taking into consideration the systemic effects of the adipokines, it is plausible that they are (partly) responsible for the above-mentioned imbalances at the hepatic level.

**Table 10:** Role of adipokines in the induction of T-cell subsets.

	**Th1**		**Th2**		**Th17**		**Treg**		**Tc**	
Leptin	↑	(146–150)	↓	(147, 151)	↑	(146, 152–155)	↓	(156, 157)	↑	(158)
			↑	(159)						
Adiponectin	↓	(160, 161)	=	(160, 161)	↓	(160–162)	↑	(163, 164)		
	↑	(165, 166)			↑	(166, 167)				
Visfatin	↑	(168)								

*Th1, T helper 1 cells; Th2, T helper 2 cells; Th17, T helper 17 cells; Treg, regulatory T cells; Tc, cytotoxic T cells*.

Thirdly, multiple other drivers could be responsible for the observed increase toward Th17 differentiation. Acetyl-CoA carboxylase 1 (ACC1), a regulatory enzyme in cellular fatty acid metabolism, was shown to be induced in obesity and to stimulate Th17 differentiation by modulating DNA binding of RORγt to target genes (169). Notably, the ACC inhibitor GS-0976 is currently under investigation in clinical trials for the treatment of NASH (NCT02856555). Similarly, hypoxia-inducible factor 1α (HIF-1α), a transcription factor that induces glycolysis and a known regulator of differentiation toward a Th17 phenotype at the expense of the Treg cell subset, is an upcoming player in obesity-associated chronic inflammation and NAFLD (170–174).

Lastly, in the past decade it has become clear that the dysbiosis in the gut microbiome is potentially involved in the pathophysiology of a myriad of diseases, including NAFLD and obesity (175). Moreover, specific commensal species have been associated with the differentiation toward Th17 and Treg cells (176–178) and Li et al. demonstrated that hepatic IL-17-producing γδ T cell homeostasis is maintained by the gut microbiota (133). Concerning the role of dysbiosis in NAFLD and obesity, several mechanisms are under investigation. One of these mechanisms is a change in the availability and composition of short-chain fatty acids (SCFAs), which are products of carbohydrate fermentation by gut microorganisms and include acetate, propionate, and butyrate (179). Compellingly, multiple studies have shown that these molecules also exert an influence on T-cell differentiation. However, whereas most studies would suggest that SCFAs induce a tolerogenic and anti-inflammatory effect (180–190), other studies provide clear evidence for an SCFA-dependent induction of Th1, Th17, and Tc phenotypes *in vitro* and *in vivo* (187–190). Interestingly, Kespohl et al. propose that these ambiguities might be explained by both the concentration of the SCFAs and the immunological milieu, inducing an anti-inflammatory response in physiologic conditions, at a low concentration and in the presence of TGFβ, while inducing a more proinflammatory response, at high SCFA concentrations and when TGFβ is scarce (181, 186). Moreover, in a recent study, Rau et al. have shown that, compared to healthy controls, NAFLD patients exhibit higher fecal SCFA levels and that a positive correlation exists between propionate and acetate levels and the Th17/Treg ratio (191). Another proposed mechanism for the involvement of dysbiosis is the increased absorption of lipopolysaccharides (LPS) as a result of an increased intestinal permeability (179). Indeed, LPS has been found to induce the proinflammatory T-cell subsets Th1 and Th17, while inhibiting the more anti-inflammatory T-cell subsets Th2 and Treg, especially when present in high concentrations (192–200). Additionally, Ghazarian et al. showed that the observed hepatic Tc accumulation and activation is driven by type I IFN responses as a result of the increased availability of bacterial products. In this sense, both SCFAs and LPS might contribute to the observed imbalances in T-cell subsets associated with NAFLD, obesity and insulin resistance.

## Concluding Remarks

Multiple T-cell subsets are involved in NAFLD pathogenesis, exerting differential effects on adiposity, insulin resistance, steatosis, hepatic inflammation, hepatic injury, and fibrosis (Figure 1). Additionally, some subsets are involved in hepatocarcinogenesis, although this was not the focus of this review. Remarkably, several subsets appear to have an attenuating effect on adiposity (Th1, Th2, Th17, Th22, and possibly NKT cells). The effect on the glucose metabolism is more diverse: Th2, Th22, and Treg cells seem to diminish insulin resistance, whereas Th1, Th17, and Tc cells have an aggravating effect. Concerning NAFLD, both Th22 and Treg cells have an overall tempering effect on multiple NAFLD features. Conversely, Tc cells seem to facilitate the development of steatosis, whereas the specific effect of Th17 cells on this feature is still under debate. Nevertheless, both Th17 and Tc cells were shown to induce more liver damage and fibrosis progression.

Importantly, the T-cell subsets involved in NAFLD and obesity are not restricted to the adaptive immune system, as NKT cells (and possibly γδT and MAIT cells) seem to be critically involved and might represent the link between the involvement of the innate and adaptive immune systems. Unfortunately, a lot of controversy about their exact role in obesity and NAFLD still exists, which represents a clear future research goal. Furthermore, it should be noted that most of the observed effects seem to be dependent of specific cytokines. These cytokines, however, are not secreted exclusively by one T-cell subset, nor even exclusively by T cells, which represents an important caveat in this research field. Additionally, it remains to be demonstrated whether the involved T-cell subsets are activated as a part of the general proinflammatory state or that rather an antigen-specific activation of distinct T-cell subsets might be involved. As reviewed thoroughly by Chng et al. in the context of obesity-related insulin resistance, there is some evidence for the latter and several mechanisms have been suggested (201). In the context of NAFLD, Sutti et al. have proposed a role for oxidative stress-related antigens (202). Moreover, as described above, some data are available about the mechanisms of adipose tissue and hepatic T-cell recruitment, proliferation and activation in the context of NAFLD and obesity. However, considering the therapeutic potential of these pathways, further exploration is certainly warranted. Lastly, one should consider that not all findings from animal studies can be readily translated to the situation in humans (203) and some clear discrepancies exist when comparing animal to human studies, particularly concerning the Treg subset. Nonetheless, the above-mentioned studies endorse a specific role for several T-cell subsets in NAFLD pathogenesis and suggest that they might be implicated in the progression from NAFL to NASH and advanced liver disease. As the latter is a major unresolved issue in NAFLD pathophysiology, further exploration of the involved mechanisms, in particular those involved in the interplay between the liver, the adipose tissue and the gut, is warranted. Moreover, multiple medical therapies that alter T-cell function, are already available for the treatment of inflammatory bowel disease, rheumatoid arthritis and psoriasis, including the combined IL-12 and IL-23 antagonist ustekinumab, the IL-17 antagonist secukinumab and the IL-6 antagonist tocilizumab. As the treatment of NASH is an important unmet medical need, the evaluation of the therapeutic potential of these agents in NAFLD might therefore be a relevant future research goal.

## Author Contributions

MVH conceptualized the review's content and wrote the manuscript. JW, WK, ED, BD, SF, and LV made contributions regarding the content and revised the manuscript.

### Conflict of Interest Statement

The authors declare that the research was conducted in the absence of any commercial or financial relationships that could be construed as a potential conflict of interest.
